# Sperm-Induced Ca^2+^ Release in Mammalian Eggs: The Roles of PLCζ, InsP_3_, and ATP

**DOI:** 10.3390/cells12242809

**Published:** 2023-12-10

**Authors:** Karl Swann

**Affiliations:** School of Biosciences, Cardiff University, Cardiff CF10 3AX, UK; swannk1@cardiff.ac.uk

**Keywords:** egg, sperm, fertilization, calcium, PLCζ, InsP_3_, ATP

## Abstract

Mammalian egg activation at fertilization is triggered by a long-lasting series of increases in cytosolic Ca^2+^ concentration. These Ca^2+^ oscillations are due to the production of InsP_3_ within the egg and the subsequent release of Ca^2+^ from the endoplasmic reticulum into the cytosol. The generation of InsP_3_ is initiated by the diffusion of sperm-specific phospholipase Czeta1 (PLCζ) into the egg after gamete fusion. PLCζ enables a positive feedback loop of InsP_3_ production and Ca^2+^ release which then stimulates further InsP_3_ production. Most cytosolic Ca^2+^ increases in eggs at fertilization involve a fast Ca^2+^ wave; however, due to the limited diffusion of InsP_3_, this means that InsP_3_ must be generated from an intracellular source rather than at the plasma membrane. All mammalian eggs studied generated Ca^2+^ oscillations in response to PLCζ, but the sensitivity of eggs to PLCζ and to some other stimuli varies between species. This is illustrated by the finding that incubation in Sr^2+^ medium stimulates Ca^2+^ oscillations in mouse and rat eggs but not eggs from other mammalian species. This difference appears to be due to the sensitivity of the type 1 InsP_3_ receptor (IP3R1). I suggest that ATP production from mitochondria modulates the sensitivity of the IP3R1 in a manner that could account for the differential sensitivity of eggs to stimuli that generate Ca^2+^ oscillations.

## 1. Introduction

**Figure 1:**
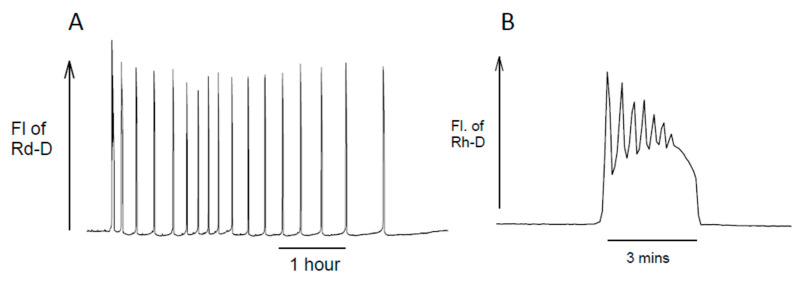
Ca^2+^ oscillations at fertilization in mouse eggs. Ca^2+^ was monitored via increases in the fluorescence of the Ca^2+^-sensitive dye Rhod2-dextran (Rh-D) that was microinjected into eggs as described previously [14]. Part (**A**) shows the spike-like increases in Ca^2+^ at fertilization, for which there are typically 10–20 Ca^2+^ spikes occurring over a period of 3–4 h. In trace (**B**) is shown a typical example of the first Ca^2+^ transient that lasts several minutes and consists of smaller oscillations on top of a larger increase.

In all studied mammalian eggs, the sperm has been shown to trigger a long-lasting series of increases in cytosolic Ca^2+^, also referred to as cytosolic Ca^2+^ oscillations [1,2,3,4]. These Ca^2+^ oscillations are both necessary and sufficient for activating development. An example recording of Ca^2+^ oscillations in a fertilizing mouse egg is shown in Figure 1A. Each transient increase in cytosolic Ca^2+^ in mammalian eggs is the result of Ca^2+^ release that is stimulated by the production of inositol 1,4,5-trisphosphate (InsP_3_), which opens the InsP_3_ receptor (IP3R) in the endoplasmic reticulum (ER).

There is substantial evidence that the Ca^2+^ oscillations at fertilization are initiated by an isoform of phospholipase C (PLC) called PLCzeta (PLCz1 or PLCζ). PLCζ is a sperm-specific protein that has been shown to cause Ca^2+^ oscillations and egg activation in mouse, human, pig, and cow eggs [5,6,7]. PLCζ is found in cytosolic sperm extracts that can cause Ca^2+^ oscillations in eggs after microinjection [7]. The presence of PLCζ in the sperm head can also explain why the direct injection of sperm into eggs, so-called intracytoplasmic sperm injection (ICSI), also triggers a series of prolonged Ca^2+^ oscillations [8,9]. PLCζ is present in sperm at a concentration that can cause Ca^2+^ oscillations in eggs, and it is localized inside the sperm in the post-acrosomal region, which is where sperm–egg fusion first occurs [7]. It is thought that the PLCζ protein diffuses into the egg in the first few minutes after sperm–egg membrane fusion.

In this review, I will specifically discuss PLCζ- and InsP_3_-induced Ca^2+^ release in mammalian eggs and the ways in which features of eggs make their InsP_3_-induced responses different from somatic cells. Two of these features involve the mechanism of action of PLCζ, and the third involves the way ATP can modulate Ca^2+^ release. This review will concentrate on the dynamics and mechanism of sperm-induced Ca^2+^ release. Other reviews are recommended for a discussion of wider aspects of Ca^2+^ homeostasis in mammalian eggs [10,11] or else the way in which Ca^2+^ oscillations stimulate meiotic resumption and embryo development [12,13].

## 2. Diffusion of PLCζ and InsP_3_ Can Explain PIP_2_ Distribution and Ca^2+^ Waves

**Figure 2:**
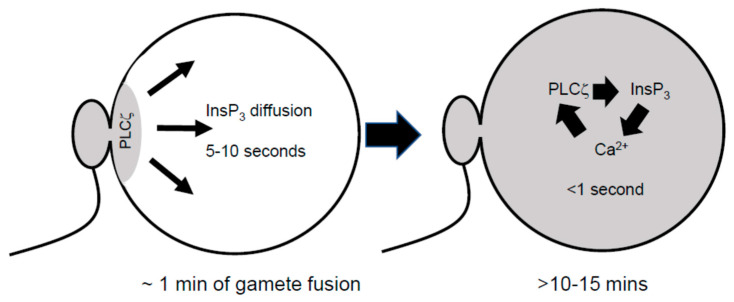
A schematic illustration of the changing pattern of Ca^2+^ waves in mouse and hamster eggs at fertilization. The initial Ca^2+^ increase is triggered by PLCζ in a restricted region of the egg near the site of sperm–egg fusion. After about 10–15 min, PLCζ will have diffused throughout the egg cytoplasm. The very fast waves are now driven by Ca^2+^-induced InsP_3_ formation from cytoplasmic PIP_2_.

The initial Ca^2+^ increase at fertilization in mouse and hamster eggs occurs as a wave of Ca^2+^ release that crosses the egg. This wave starts from the point of sperm–egg fusion and travels to the opposite side of the egg [3,15]. The time taken for the initial Ca^2+^ wave in these eggs is about 5–10 s [15]. In subsequent Ca^2+^ transients, the waves become increasingly rapid, such that after about 15 min, the waves cross the egg in ~1 s and the initiation point is near the cortex, but it varies from one transient to the next [15] (Figure 2).

When initial Ca^2+^ waves occur, PLCζ will be restricted to the area of sperm–egg fusion. Hence, the initial waves propagate from where PLCζ is concentrated into a cytoplasm that is mostly devoid of PLCζ (see Figure 2). PLCζ is a 70 kDa protein, and the diffusion time for proteins of around this molecular weight across the mouse egg is about 10 min [16]. This means that after ~10 min, PLCζ is expected to have diffused throughout the egg cytoplasm. The spread of PLCζ is coincident with later Ca^2+^ oscillations which have a rapid rising phase (<1 s) and are known to comprise a Ca^2+^ wave that travels at 50–100 μm per second (Figure 2). This shift in the pattern of waves has been shown at fertilization in both mouse and hamster eggs [3,15]. The very rapid Ca^2+^ waves that take over after 10–20 min in either type of egg imply the existence of a positive feedback loop of Ca^2+^ release. The mechanism of this positive feedback loop helps explain the unusual nature of phosphatidylinositol 1,4,5-bisphoshate (PIP_2_) distribution in mammalian eggs.

We know that PLCζ hydrolyses PIP_2_ in eggs to generate InsP_3_, which causes Ca^2+^ release [7], but the source of PIP_2_ is less obvious. Like most other cell types, PIP_2_ is detected in the plasma membrane of mouse eggs. However, unlike other cell types, much of the PIP_2_ in mouse eggs is localized in small vesicles that are dispersed throughout the cytoplasm [17,18]. The identity of these vesicles is not clear, but they are likely to be Golgi-derived [18]. The reason why PIP_2_ in eggs is present in such cytoplasmic vesicles has been unclear, but this localization may be necessary because of a fundamental feature of the mechanism of Ca^2+^ oscillations in eggs.

There are two classes of models for explaining cytosolic Ca^2+^ oscillations in cells, and the models differ in the nature of the positive feedback loop [19,20]. One set of models relies upon the ability of the IP3R to open in response to InsP_3_, and this opening is enhanced by increasing concentrations of Ca^2+^. The Ca^2+^-enhanced Ca^2+^ release operates for the initial phase of Ca^2+^ release, but IP3R then closes when Ca^2+^ levels rise to approach the micromolar range. This class of models involves the positive feedback of Ca^2+^ on theIP3R, and they are therefore referred to as IP3R-based models [19,20]. It has been shown that mouse and human eggs predominantly contain the type 1 IP3R (IP3R1) [21,22], which shows a bell-shaped response to Ca^2+^ release when stimulated by InsP_3_; hence, Ca^2+^ can act to enable positive and negative feedback upon Ca^2+^ release in eggs [23].

The other set of models involve InsP_3_-induced Ca^2+^ release, with Ca^2+^ then stimulating further PLCζ activity, and this leads to more InsP_3_ and hence more Ca^2+^ release. These are the so-called regenerative InsP_3_ production models. PLCζ is known to be stimulated by Ca^2+^ at concentrations from around 100 nM to 1 μM [24,25]. PLCζ contains four EF hand domains which are essentially for this high sensitivity to Ca^2+^ [25]. Hence, PLCζ can provide the basis for such a positive feedback loop of InsP_3_ production in eggs [24,25]. It should be noted that when InsP_3_ concentrations are high, the inhibitory effect of Ca^2+^ on the IP3R is effectively switched off; hence, Ca^2+^ concentrations can continue to increase to above 1 μM [26]. Either of these two models of oscillation can operate in mammalian eggs and could vary depending upon the stimulus. The critical question is which model applies to Ca^2+^ oscillations at fertilization or in response to PLCζ.

Fortunately, it is possible to distinguish which mechanism is operating during Ca^2+^ oscillations in a relatively simple and definitive manner. The mechanism of InsP_3_-dependent Ca^2+^ oscillations can be established by applying a pulse of InsP_3_ during oscillations. This can be carried out in live cells via the photo-release of caged InsP_3_ [18]. With receptor-based models of regenerative Ca^2+^ release, a pulse of InsP_3_ always causes an increase in the frequency of Ca^2+^ oscillations [19]. In contrast, with Ca^2+^-induced regenerative InsP_3_ formation models, a pulse of InsP_3_ leads to an immediate Ca^2+^ transient, and this causes a resetting of the phase of oscillations [19]. In practice, what one sees with this model is that a pulse of InsP_3_ causes a single Ca^2+^ transient, and the next Ca^2+^ transient is seen after same interval as all other oscillations, starting from this single transient. Hence, there is no increase in frequency (e.g., Figure 2 in [18]). This means that one sees two very different types of behavior depending upon whether oscillations are based on the IP3R alone or whether they are based upon regenerative InsP_3_ production. The difference is fundamental to way InsP_3_ works and does not depend upon the details of the model [19]. It has previously been used to distinguish the different types of Ca^2+^ oscillations in some somatic cells [19].

We have found that when InsP_3_ is photo-released in eggs that are oscillating in response to PLCζ, a pulse of InsP_3_ consistently causes an immediate Ca^2+^ transient and a resetting of the phase of Ca^2+^ oscillations, with no increase in frequency (see Figure 2 in Sanders et al. [18]). This shows that when PLCζ is distributed throughout the cytoplasm, Ca^2+^ oscillations occur with an increasing phase that depends upon Ca^2+^-induced InsP_3_ production. Interestingly, this is not true for all types of Ca^2+^ oscillations in eggs. For example, mouse eggs can also be induced to undergo Ca^2+^ oscillations via incubation in a Sr^2+^ medium. With these Sr^2+^-induced-Ca^2+^ oscillations, which occur without InsP_3_ generation, an artificially applied photo-release pulse of InsP_3_ increases the frequency of Ca^2+^ oscillations [18]. Hence, Sr^2+^ causes Ca^2+^ oscillations in mouse eggs by acting upon the IP3R1 alone, which is consistent with data showing that there are direct stimulatory effects of Sr^2+^ on IP3R1s in cerebella microsomes [27]. There are clearly two mechanisms of Ca^2+^ oscillations that can exist in mouse eggs, one for PLCζ and another for Sr^2+^. Significantly, they demonstrate that PLCζ causes Ca^2+^ oscillations via a positive feedback mechanism based upon Ca^2+^-induced InsP_3_ generation. This means there needs to be a regenerative rise in InsP_3_ that accompanies the rapid Ca^2+^ waves that cross the egg in ~1 s.

The rapid generation of InsP_3_ from PIP_2_ hydrolysis during the rising phase of Ca^2+^ increase raises an important issue for large spherical cells like mammalian eggs. In most somatic cell types, PIP_2_ is predominantly, if not entirely, found in the plasma membrane. Such cells are often small or flat, and InsP_3_ can diffuse from the plasma membrane to fill the cytoplasm within a few seconds. However, in eggs, if all PIP_2_ hydrolysis did occur at the plasma membrane, then InsP_3_ diffusion would set a limit on the propagating speed of the Ca^2+^ wave.

Determining diffusion coefficients inside cells is not trivial, but the most recent studies suggest a diffusion coefficient of ~100 μm^2^/s [28]. The diffusion range for a molecule is approximately √2 Dt, where ‘D’ is the diffusion coefficient and ‘t’ is the time scale. Given this, we can estimate that the timescale for InsP_3_ to travel the 35 μm to the center of a mouse egg will be around 6 s. This is too slow to explain the fast Ca^2+^ waves that cross the egg in <1 s. Hence, plasma membrane PIP_2_ hydrolysis will be unable to support the Ca^2+^ wave that is observed [15]. For the rapid Ca^2+^ wave to occur, the source of InsP_3_ production must be much closer to the site of Ca^2+^ release.

We have modeled Ca^2+^-induced InsP_3_ production during the rising phase of a Ca^2+^ spike in mouse eggs and estimate that the source of InsP_3_ needs to be within a few microns of the IP3Rs [18]. By labeling intact mouse eggs with a fluorescent probe for PIP_2_, we found that the PIP_2_ vesicles are dispersed about 2 microns apart from one another (see Figure 5 in reference [18]). The ER is distributed throughout the egg cytoplasm, so it is likely to be approximately the same distance away from the PIP_2_ vesicles. Hence, the unusual distribution of PIP_2_ in cytoplasmic vesicles is consistent with, if not essential for explaining, the known dynamics of oscillations and waves in mouse and hamster eggs. The idea that InsP_3_ is generated from cytoplasmic PIP_2_ vesicles is also consistent with the finding that there is no detectable PIP_2_ hydrolysis in the plasma membrane of mouse eggs during fertilization [17].

## 3. The Dynamics of InsP_3_ at Fertilization

The model of PLCζ-induced Ca^2+^ oscillations in eggs predicts that InsP_3_ should oscillate in phase with Ca^2+^. Two studies have reported measurements of InsP_3_ during Ca^2+^ oscillations in mouse eggs. The initial study detected InsP_3_ oscillations in PLCζ-injected mouse eggs as predicted, but only with higher concentrations of PLCζ, and there were no InsP_3_ increases detected during fertilization [29]. This may have been an issue with the sensitivity of the InsP_3_ indicator used. A more recent study used a more sensitive InsP_3_ indicator called IRIS-2.3_TMR_ and detected InsP_3_ increases during fertilization [30]. It was found that there was a small and monotonic increase in InsP_3_ during the initial Ca^2+^ transients, but then distinct oscillations in InsP_3_ were seen after about 20 min into the series of Ca^2+^ transients [30].

This shift in pattern is consistent with the diffusion time for PLCζ discussed above. In the first 10–20 min, PLCζ will be localized around the site of sperm fusion, and oscillations will be mainly dependent upon InsP_3_ diffusion, leading to oscillations dependent upon IP3R stimulation. After 10–20 min, PLCζ will have diffused throughout the egg, and the oscillations are then dependent upon Ca^2+^-induced InsP_3_ production, with InsP_3_ oscillating in synchrony with Ca^2+^ oscillations (Figure 2). Hence, these experiments are consistent with the idea that an IP3R-based model is operative for the initial Ca^2+^ transients at fertilization but that after 20 min, the mechanism of regenerative InsP_3_ production from PIP_2_ vesicles starts to dominate, and this second mechanism accounts for most of the Ca^2+^ transients that can occur for the 2–4 h after sperm–egg fusion (Figure 2).

The Ca^2+^ dependency of PLCζ may also be important in setting the frequency of Ca^2+^ oscillations over the 3–4 h of the fertilization response. This is because the inter-spike level of Ca^2+^ will affect the amount of InsP_3_ that is produced. When fertilizing eggs, or when eggs injected with PLCζ are placed into a Ca^2+^-free medium, the Ca^2+^ oscillations slow down or stop [31,32]. It has also been shown that the knockout of TRM7 and Cav3.2 Ca^2+^ channels in eggs lead to a reduction in the frequency of Ca^2+^ oscillations at fertilization [33]. These data clearly show that Ca^2+^ influx is required to maintain Ca^2+^ oscillations. This result is often interpreted as showing that Ca^2+^ influx is important for maintaining Ca^2+^ store content and that the store content plays a role in the timing of each Ca^2+^ transient. It is clear that Ca^2+^ will decrease in the ER during each Ca^2+^ transient in the cytosol, but the rapid cessation of Ca^2+^ oscillations in a Ca^2+^-free medium may not be due to ER depletion as such. This is because reducing Ca^2+^ influx into eggs will also reduce the level of cytosolic Ca^2+^, and this will then decrease the stimulation of Ca^2+^-dependent InsP_3_ generation from PLCζ. Hence, in a Ca^2+^-free medium, there will be both a lower ER Ca^2+^ concentration and a lower cytosolic Ca^2+^ concentration.

The relative importance of Ca^2+^ store content versus cytosolic Ca^2+^ level can be distinguished via a simple experiment using the ER Ca^2+^ pump inhibitor thapsigargin [18]. Applying low concentrations of thapsigargin can be used to reduce the Ca^2+^ store content whilst increasing cytosolic Ca^2+^ levels. If mouse eggs are induced to undergo Ca^2+^ oscillations via the injection of PLCζ, the oscillations can be slowed down, or stopped, by placing them in a Ca^2+^-free medium. When low concentrations of thapsigargin are then added to these eggs (which are still in a Ca^2+^-free medium), Ca^2+^ oscillations can be restarted, and the oscillations can continue for more than 1 h [18]. During these oscillations, there is a small increase in cytosolic Ca^2+^ levels and a slight depletion of Ca^2+^ stores, as shown by a smaller amplitude of Ca^2+^ spikes. However, the data are remarkable in that they show that mouse eggs can undergo Ca^2+^ oscillations for more than an hour in a Ca^2+^-free medium if cytosolic Ca^2+^ is elevated. We know that the activity of PLCζ is very sensitive to increases in Ca^2+^ around the 100–500 nM range [24] and that thapsigargin causes a small increase cytosolic Ca2+ within this range [17]. Hence, the simplest explanation for this data is that the ability of cytosolic Ca^2+^ to stimulate PLCζ can play a key role in triggering each Ca^2+^ spike and that the Ca^2+^ store content may be less significant. This result also implies that it requires more than an hour of oscillations for Ca^2+^ stores to become depleted of Ca^2+^ to the point that no more release occurs.

## 4. Models of Ca^2+^ Oscillations in Eggs

There are many mathematical models of intracellular Ca^2+^ oscillations in somatic cells [19,20]. We have a good understanding of how the sperm causes Ca^2+^ release in mammalian eggs, but there are no specific mathematical models for mammalian eggs, and some features have yet to be explained in the context of existing generic models of Ca^2+^ oscillations.

One distinctive feature in mouse eggs, shown in Figure 1B, is that the first Ca^2+^ spike usually has multiple spikes on top of a larger, longer Ca^2+^ increase. This was seen in some of the first Ca^2+^ recordings of Ca^2+^ oscillations, which used aequorin to measure Ca^2+^, and we see it in most of our recordings using Ca^2+^ dyes at fertilization and with PLCζ [1,7]. These small oscillations on top of the first spike are a feature of some models, but we do not know how they occur in eggs.

In addition, we find that there is often a transient increase in the frequency of Ca^2+^ oscillations at about an hour after sperm–egg fusion (Figure 1A). We see this using microinjected dextran-linked Ca^2+^ dyes to measure Ca^2+^, but we did not see with earlier recordings using AM-loaded Ca^2+^ dyes which may be less reliable because they are associated with formaldehyde generation [34]. Most significantly, it is not clear how each Ca^2+^ increase is terminated. We can explain the regenerative rise in terms of Ca^2+^ stimulating PLCζ [18], but the nature of the negative feedback that decreases Ca^2+^ levels may require some rethinking. Some models of Ca^2+^ oscillations invoke the complete emptying of Ca^2+^ stores to terminate Ca^2+^ release, but this does not occur during each Ca^2+^ transient in mouse eggs [18,32]. Most models invoke a Ca^2+^-induced desensitization of the IP3R to terminate Ca^2+^ release [19,20]. However, when InsP_3_ concentrations are high, which is expected with PLCζ, Ca^2+^ is not effective in closing the IP3R [26]. Ca^2+^-induced desensitization of IP3Rs may not occur physiologically for most Ca^2+^ transients in fertilizing mammalian eggs.

## 5. PLCζ and Other Sperm Factors

PLCζ has been shown to cause Ca^2+^ oscillations when injected as RNA or protein into mouse, human, pig, or cow eggs. Such studies have involved many different research groups; hence, there are multiple independent replications of the ability of PLCζ to cause Ca^2+^ oscillations. However, there is also evidence that there might be another sperm factor in mammalian fertilization. This is because when mouse eggs are fertilized by PLCζ KO sperm, there are still somewhere between one and four Ca^2+^ spikes that occur about an hour after sperm–egg fusion [9,35]. It is entirely unclear how sperm can trigger these Ca^2+^ oscillations. It is possible that the sperm could introduce a second factor into the egg that causes InsP_3_ production and Ca^2+^ release.

There are some sperm factor candidates that have been proposed as possible alternatives or additional factors to PLCζ. These other sperm factors include a protein called PAWP and an extramitochondrial form of citrate synthase [36,37,38]. Both proteins have been suggested to be capable of causing Ca^2+^ release and egg activation in mammalian eggs. We carried out a series of experiments microinjecting PAWP RNA or PAWP protein into mouse eggs. We also tested the effects peptides of from PAWP that were reported to block Ca^2+^ oscillations at fertilization. We could not reproduce any of the original findings on PAWP and found no evidence that it causes Ca^2+^ release [39,40]. We have also injected citrate synthase (which is commercially available) into mouse eggs and found no sign of Ca^2+^ release. Consequently, a problem with these other candidate sperm factors is that the primary studies are not readily reproducible. Until independent laboratories can reproduce the claimed effects of PAWP or citrate synthase, it is difficult to assess their role in fertilization. Such failures to reproduce key observations contrast with the numerous independent studies on the effects of PLCζ.

Whatever the nature of a putative second sperm factor, there are some characteristics of Ca^2+^ release in eggs fertilized by PLCζ KO sperm that need to be explained. For example, when PLCζ KO sperm are injected into mouse eggs (as in ICSI), there are no Ca^2+^ oscillations [9,35]. However, as noted above, PLCζ KO sperm cause 1–4 Ca^2+^ spikes after sperm–egg fusion in IVF [9]. Hence, the second factor works in IVF but not in ICSI. It is not known why the putative second factor only operates during normal IVF. Also, in IVF with PLCζ KO sperm, there is always a distinct delay of about 1 h between sperm–egg fusion and the initiation of Ca^2+^ oscillations. It is again unclear why it should take about one hour for a putative second factor to trigger Ca^2+^ release. Hopefully, the identification of a ‘second sperm factor’ will provide an explanation for these characteristics.

## 6. ATP and the Sensitivity of Ca^2+^ Release in Eggs

One feature of Ca^2+^ release in mammalian eggs that is rarely discussed is the way that eggs from different species differ in the ability to undergo Ca^2+^ oscillations. This was evident from early studies on hamster and mouse eggs. For example, inserting a micropipette to cause a sustained injection of InsP_3_ causes a heavily damped series of Ca^2+^ oscillations in hamster eggs that typically stop after 10–15 min [41]. However, the same type of experiment with the sustained injection of InsP_3_ into mouse eggs leads to undamped and prolonged Ca^2+^ oscillations, even with tenfold lower concentrations of InsP_3_ [42]. We later found that mouse eggs are much more sensitive to human PLCζ than human eggs [43]. The difference in sensitivity to sperm PLCζ is at least 30-fold, which is more than can be accounted for by the size difference between mouse and human eggs.

The difference in sensitivity between eggs of different species is most evident with Sr^2+^ which, as noted above, stimulates the IP3R1 to cause Ca^2+^ release. Incubation in a Sr^2+^ medium is very effective and reliable in causing prolonged Ca^2+^ oscillations in mouse eggs and rat eggs [4,18,44,45]. However, under the same conditions, human eggs do not show any Ca^2+^ release in response to a Sr^2+^ medium [44,46].

It is not clear why this difference exists because mouse and human eggs have the same IP3R1 and the same plasma membrane TrV3 channels that allow Sr^2+^ to enter the egg [44,47,48]. Despite the simplicity and significance of the experiment, it is notable that there are no reports for Sr^2+^-induced Ca^2+^ oscillations in pig, cow, or hamster eggs. These data suggest that mouse and rat eggs have much more sensitive Ca^2+^ release than eggs from humans and hamsters and probably compared to eggs from pigs and cattle.

It has not been clear why eggs from different mammalian species differ in their sensitivity to PLCζ, InsP_3_, and Sr^2+^, but the underlying difference appears to be due to the IP3R. We have tested the sensitivity of eggs using caged InsP_3_ and UV light to deliver precise pulses of InsP_3_. We found that mouse eggs are about 10 times more sensitive to InsP_3_-induced Ca^2+^ release than human eggs [46]. The implication is that something in mouse eggs is sensitizing IP3R1s in a way that is not found in human eggs. There may be some subtle difference in the structure of the IP3R1 between mouse and human eggs, but it is not known what this might be. The IP3R can be modulated by cycle cell protein kinases [11], but it is not obvious why this would be different between mouse and human eggs since they are both arrested at the metaphase of second meiosis. We recently presented data that suggest the relevant factor that regulates the IP3Rs in mammalian eggs is the concentration of ATP. 

Most somatic cells express combinations of type 2 or 3 IP3Rs, but all mammalian eggs appear to predominantly express the IP3R1. The three different IP3R subtypes show some differences in sensitivity to InsP_3_ and Ca^2+^ [49]. However, the IP3R1 is markedly different from the IP3R2 and IP3R3 in the way it is regulated by ATP. The IP3R1 is stimulated to increase its open probability in response to ATP, and this contrasts with the type 3 IP3R, which requires ~10-fold higher concentrations to be opened by ATP, and the type 2 IP3R, which is not modulated by ATP [49]. Hence, mammalian eggs, that express high levels of IP3R1s, may be sensitive to ATP in a way that is not seen in many other cell types.

The regulation of the IP3R1 by ATP is allosteric and does not involve ATP hydrolysis. In fact the form of ATP that has been shown to stimulate the IP3R1 in frog oocytes is the Mg^2+^-free form, or ATP^4−^, which is different from the MgATP^2−^ that provides the energy for ion pumps and other energy-consuming processes [50]. The half-maximal concentration of ATP^4−^ needed to activate Xenopus oocyte IP3Rs is ~270 μM, which is consistent with other studies that suggest a range of 100 to 400 μM of ‘free’ ATP^4−^ for stimulating the IP3R1 [49,50,51]. About 95% of the ATP in the cytosol is bound to Mg^2+^ as MgATP^2−^. However, if the total concentration of ATP is around 3.3 mM for mouse eggs [46] and we take the free Mg^2+^ concentration as 1 mM, which is reported for mammalian cells [52], then the ATP^4−^ concentration in mouse eggs will be ~200 μM (calculated using Maxchelator software [53]). Hence, the ATP^4−^ concentration in mouse eggs is within the range in which it could physiologically modulate the opening of the IP3R1. The idea that ATP^4−^ physiologically modulates the IP3R1 was previously suggested for frog oocytes [50].

We have found that the concentration of total ATP in mouse eggs is higher than in human eggs. Mouse eggs have a total ATP concentration of around 3.3 mM, whereas we found that it was 1.4 mM for human eggs, which is equivalent to about 90 μM of ATP^4−^ [46]. The difference in total ATP concentrations between mouse and human eggs is evident from some previous studies but may have gone unnoticed because of the practice of reporting ATP in picomoles/egg rather than in metric units. Reports state that human eggs have an ATP/egg about twice that of mouse eggs, but the human egg is nearly five times the volume of a mouse egg, so human eggs actually have a lower total ATP concentration [54,55]. Interestingly, data from one study reported that mouse eggs have a total ATP concentration about three times higher than that of hamster eggs [56]. Hence, it is likely that the homeostatic or ‘set concentration’ of total ATP and hence ATP^4−^ may be significantly higher in mouse eggs compared to eggs from many other mammalian species. These differences may not have a large impact on the need for the energetic use of MgATP, for example, in pumping Ca^2+^ into the ER, because the energy available depends upon the logarithm of the concentration ratio of MgATP over MgADP plus phosphate.

**Figure 3:**
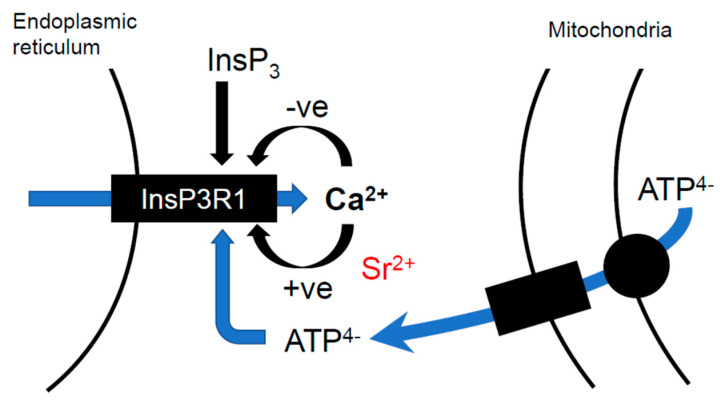
A schematic diagram of the way ATP^4−^ may regulate the IP3R1 in eggs. The IP3R1 in the ER membrane can respond to Ca^2+^ at two sites, one inhibitory and one stimulatory. InsP_3_ binding inhibits the inhibitory site (−ve) to stimulate Ca^2+^ release [26,60]. ATP^4−^ increases the Ca^2+^ sensitivity of the stimulatory site (+ve) to promote Ca^2+^ release [50]. Sr^2+^ appears to promote IP3R1 opening by binding predominantly to the stimulatory site [27,60]. Mitochondria export ATP into the cytosol in the form of ATP^4−^, which will rapidly form a complex with Mg^2+^, but ATP^4−^ may be more concentrated in regions close to the mitochondrial outer membrane.

**Figure 4:**
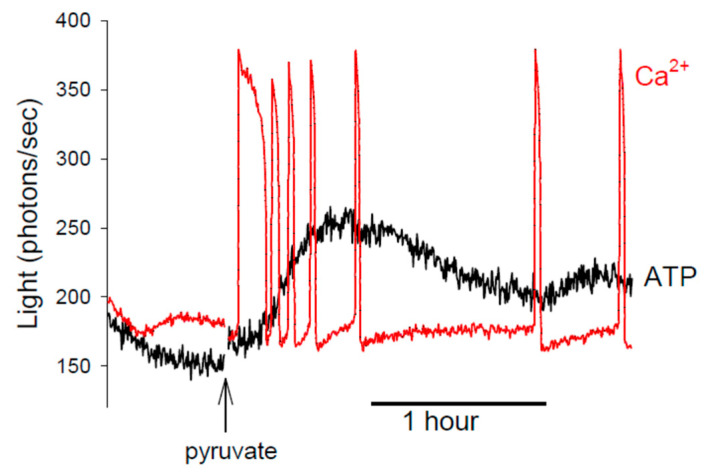
A recording of Ca^2+^ and ATP changes in mouse eggs incubated in a medium containing 5 mM Sr^2+^, as described and presented previously in Storey et al. [46]. The Ca^2+^ changes are measured with a fluorescent dye and shown in red; the ATP changes are monitored using the luminescence of luciferase and shown in black. In the initial part of the recording, the eggs are in a medium that lacks any metabolites; it has low ATP, and no Ca^2+^ oscillations are seen. After ~40 min, pyruvate (1 mM) is added to the medium containing the eggs. This leads to an increase in ATP and the triggering of Ca^2+^ oscillations.

We have found that the concentration of total ATP appears to have a significant effect on Sr^2+^-induced Ca^2+^ release in mouse eggs. One can reduce total ATP levels by removing metabolites, such as pyruvate, from the culture medium [57,58]. When mouse eggs are placed in a medium containing Sr^2+^ but with no pyruvate, they have low ATP and fail to show any Ca^2+^ oscillations, which is unusual for mouse eggs [46]. When pyruvate is added back to the medium, the ATP levels increase, and Ca^2+^ oscillations are triggered (see the example in Figure 4) [46]. These data clearly suggest that the concentration of ATP has a significant role in modulating the sensitivity of Ca^2+^ release in eggs. It is also worth noting that most of the Ca^2+^ oscillations in these pyruvate-Sr^2+^ experiments occurred during the rising phase of the ATP (Figure 3) [46]. This may be surprising because there are fewer Ca^2+^ oscillations after ATP reaches its peak. However, it should be noted that firefly luciferase responds to MgATP and not to ATP^4−^, which is probably the key modulator of IP3R1s. ATP^4−^ will be produced at the greatest rate during the rising phase of MgATP because mitochondria uniquely produce ATP^4−^, which they export into the cytosol via the adenine nucleotide exchanger [59]. Since mitochondria are often within 50 nm of the endoplasmic reticulum, it is possible that in responding to ATP^4−^, the IP3R1 is more sensitive to the activity of mitochondria rather than the concentration of total ATP, even though the two are obviously related (see Figure 3) [23,58].

These studies of ATP in mouse eggs imply that human eggs, and possibly eggs from domesticated animals, could be induced to undergo Ca^2+^ oscillations in response to a Sr^2+^ medium if a suitable mechanism could be found to promote mitochondrial activity. Increasing ATP production in eggs is not trivial since adding extra substrates, such as pyruvate, above the level in a standard culture medium, does not increase the ATP concentration [61]. Egg mitochondria may be difficult to stimulate because they may be ‘downregulated’ in their activity as part of a mechanism to reduce reactive oxygen species production, which could otherwise damage mitochondrial DNA [61,62]. However, it is worth noting that that there is an increase in ATP concentration at fertilization in mouse eggs. This is caused in part by the Ca^2+^ stimulation of mitochondrial dehydrogenases [14,58]. The increase occurs in two phases, and the second phase of increase occurs about 1 h after the start of Ca^2+^ oscillations [14,57]. The ATP concentration increase is about twofold, so even the mouse egg, with its relatively high ATP concentration, has the capacity to increase the level of ATP given an appropriate stimulus (Figure 4).

## 7. Conclusions

Many of the studies of Ca^2+^ release in eggs rely upon research on Ca^2+^ signaling in somatic cells. I have highlighted important features of Ca^2+^ release in mammalian eggs that make them different to somatic cells. Eggs are very large spherical cells, and most eggs have a Ca^2+^ wave at fertilization that propagates throughout the cytoplasm. I suggest that the fast Ca^2+^ waves at fertilization in mammalian eggs require that PIP_2_ is distributed throughout the cytoplasm. Interestingly, substantial amounts of PIP_2_ appear to be associated with yolk granules in the cytoplasm of eggs from frogs and sea urchins [63,64]. The Ca^2+^ wave in these and other non-mammalian species is not as fast, but some mathematical models of invertebrate eggs suggest that the generation of InsP_3_ from intracellular sources may also be needed to generate these Ca^2+^ waves [65].

Another special feature of eggs is that they all appear to express a version of the type 1 IP3R, and eggs from all species contain a high density of mitochondria. There is now evidence that the modulation of IP3R1s can occur by mitochondrially generated ATP^4−^ in mouse eggs. The effects of ATP^4−^ may be a more widespread phenomenon, and it could affect the ability of eggs from different species to generate Ca^2+^ waves or oscillations in response to sperm at fertilization.
